# Amino acid sequence associated with bacteriophage recombination site helps to reveal genes potentially acquired through horizontal gene transfer

**DOI:** 10.1186/s12859-020-03599-y

**Published:** 2020-07-24

**Authors:** Maria A. Daugavet, Sergey V. Shabelnikov, Olga I. Podgornaya

**Affiliations:** 1 Institute of Cytology, St. Petersburg, Russia 194064; 2 School of Biomedicine, Far Eastern Federal University, Vladivostok, Russia 690090; 3 Department of Cytology and Histology, St. Pb State University, St. Petersburg, Russia 199034

**Keywords:** Bacteriophages, Recombination site, Protein domains, Horizontal gene transfer, Cysteine-rich repeats

## Abstract

**Background:**

Horizontal gene transfer, i.e. the acquisition of genetic material from nonparent organism, is considered an important force driving species evolution. Many cases of horizontal gene transfer from prokaryotes to eukaryotes have been registered, but no transfer mechanism has been deciphered so far, although viruses were proposed as possible vectors in several studies. In agreement with this idea, in our previous study we discovered that in two eukaryotic proteins bacteriophage recombination site (AttP) was adjacent to the regions originating via horizontal gene transfer. In one of those cases AttP site was present inside the introns of cysteine-rich repeats. In the present study we aimed to apply computational tools for finding multiple horizontal gene transfer events in large genome databases. For that purpose we used a sequence of cysteine-rich repeats to identify genes potentially acquired through horizontal transfer.

**Results:**

HMMER remote similarity search significantly detected 382 proteins containing cysteine-rich repeats. All of them, except 8 sequences, belong to eukaryotes. In 124 proteins the presence of conserved structural domains was predicted. In spite of the fact that cysteine-rich repeats are found almost exclusively in eukaryotic proteins, many predicted domains are most common for prokaryotes or bacteriophages. Ninety-eight proteins out of 124 contain typical prokaryotic domains. In those cases proteins were considered as potentially originating via horizontal transfer. In addition, HHblits search revealed that two domains of the same fungal protein, Glycoside hydrolase and Peptidase M15, have high similarity with proteins of two different prokaryotic species, hinting at independent horizontal gene transfer events.

**Conclusions:**

Cysteine-rich repeats in eukaryotic proteins are usually accompanied by conserved domains typical for prokaryotes or bacteriophages. These proteins, containing both cysteine-rich repeats, and characteristic prokaryotic domains, might represent multiple independent horizontal gene transfer events from prokaryotes to eukaryotes. We believe that the presence of bacteriophage recombination site inside cysteine-rich repeat coding sequence may facilitate horizontal genes transfer. Thus computational approach, described in the present study, can help finding multiple sequences originated from horizontal transfer in eukaryotic genomes.

## Background

As a general rule, genetic material is inherited by an offspring from its parent. This type of gene transfer is called vertical. Another way of gene flow is horizontal gene transfer (HGT), which means the acquisition of DNA from non-related species [1]. This phenomenon is widely accepted for prokaryotes [2, 3]. Mechanisms of HGT in prokaryotes are well studied and include transformation, conjugation and transduction [4]. Multiple cases of gene transfer from prokaryotes to eukaryotes have also been registered [5, 6]. For instance, one of the mechanisms of DNA transfer from *Agrobacterium* species to its plant host involves type IV secretion systems (T4SSs) of bacteria [7]. It was also shown that bacteria *Escherichia coli* [8] can deliver DNA via conjugation-like mechanism to cultured eukaryotic cells under artificial conditions [9, 10]. Nevertheless, no such mechanisms as are common between prokaryotes have been discovered so far for metazoans in nature. Yet the importance of HGT for latter may be quite significant [7, 11]. For example, the acquisition of the lysyl oxidase enzyme, one of the metazoan synapomorphy, might have involved a prokaryote source [12]. HGT is also suspected to contribute to the fast Cambrian radiation of Metazoa [13, 14]. Virus involvement as a carrier of foreign DNA [15] was proposed for many cases of horizontal transfer of transposons [16] and protein coding sequences [17–19]. Phage lambda is believed to be involved in DNA exchange between bacteria and human somatic cells [20]. *Escherichia coli* PK1A2 bacteriophage was shown to penetrate into eukaryotic neuroblastoma cells under experimental conditions [21], albeit nuclear delivery of DNA was not detected in that study. For some viruses, the presence of the nuclear localization signals was shown in their terminal proteins. Those nuclear localization signals proved to be functional and to facilitate gene delivery into the eukaryotic nucleus [22].

Cases of HGT from prokaryotes to eukaryotes are common among Fungi [23–25] and unicellular organisms [26–29]. They are less frequent among metazoans, however, with some groups more prone to HGT from prokaryotes than others. Increased HGT susceptibility may be due to asexual reproduction [30] and/or to the contact of gametes with the environment. Multiple cases of HGT were reported, for example, in nematodes [31–33], rotifers [34–36] and cnidarians [37]. Tunicates, a basal chordate group, are exceptional in their use of various ways of asexual reproduction [38, 39]. Previous studies revealed two proposed cases of HGT from prokaryotes to tunicates [40, 41]. The first one is the cellulose-synthase gene of ascidians, which was gained from bacterial donor *Streptomyces* sp. [40]. The second case involves a possibly chimeric protein rusticalin from ascidian *Styela rustica*, in which the coding sequence of the C-terminal domain might have been inherited from bacteriophage A500 [41].

Rusticalin was described as a hyalinocytes-specific protein of *Styela rustica*. The only discernible homologues of rusticalin were found in basal chordates, corals, and placozoans. According to the predicted features, based on the sequence analysis, rusticalin should consist of two distinct regions, the N-terminal domain and the C-terminal domain. The N-terminal domain comprises two cysteine-rich repeats and shows remote similarity to the tick carboxypeptidase inhibitor and also to β-defensin antibacterial peptides. The C-terminal domain, on the other hand, shares significant sequence similarity with bacterial MD peptidases and bacteriophage A500 L-alanyl-D-glutamate peptidase. Thus, the N-terminal domain of rusticalin comprises two cysteine-rich repeats of supposedly eukaryotic origin, and C-terminal domain potentially has a prokaryotic origin [41]. The coding region of the N-terminal domain contains introns with possible bacteriophage recombination sites (AttP) hidden inside, which means that C-terminal domain is adjacent to a possible AttP(s). Both sequence similarity and the presence of a putative bacteriophage recombination site support the hypothesis of the C-terminal domain coming from bacteriophage genome.

In another example of HGT in ascidians, the coding region of the cellulose-synthase catalytic domain also neighbored a sequence similar to bacteriophage recombination site. Based on these results, we proposed that a cellulose synthase catalytic subunit was acquired through the similar mechanism, involving bacteriophage as a vector. Thus, our previous work suggested a possible HGT mechanism involving bacteriophage insertion in at least two cases of transfer [41]. In the present study we aimed to find additional cases of HGT using the sequence of bacteriophage recombination site. Since the nucleotide sequence of AttP is too short, we used instead the amino acid sequence (cysteine-rich repeats) harboring AttP as a possible marker of transfer events inside eukaryotic chromosomes. About a hundred of proteins that possibly originated through HGT were found in this way.

## Results

### Proteins containing cysteine-rich repeats

**Fig. 1:**
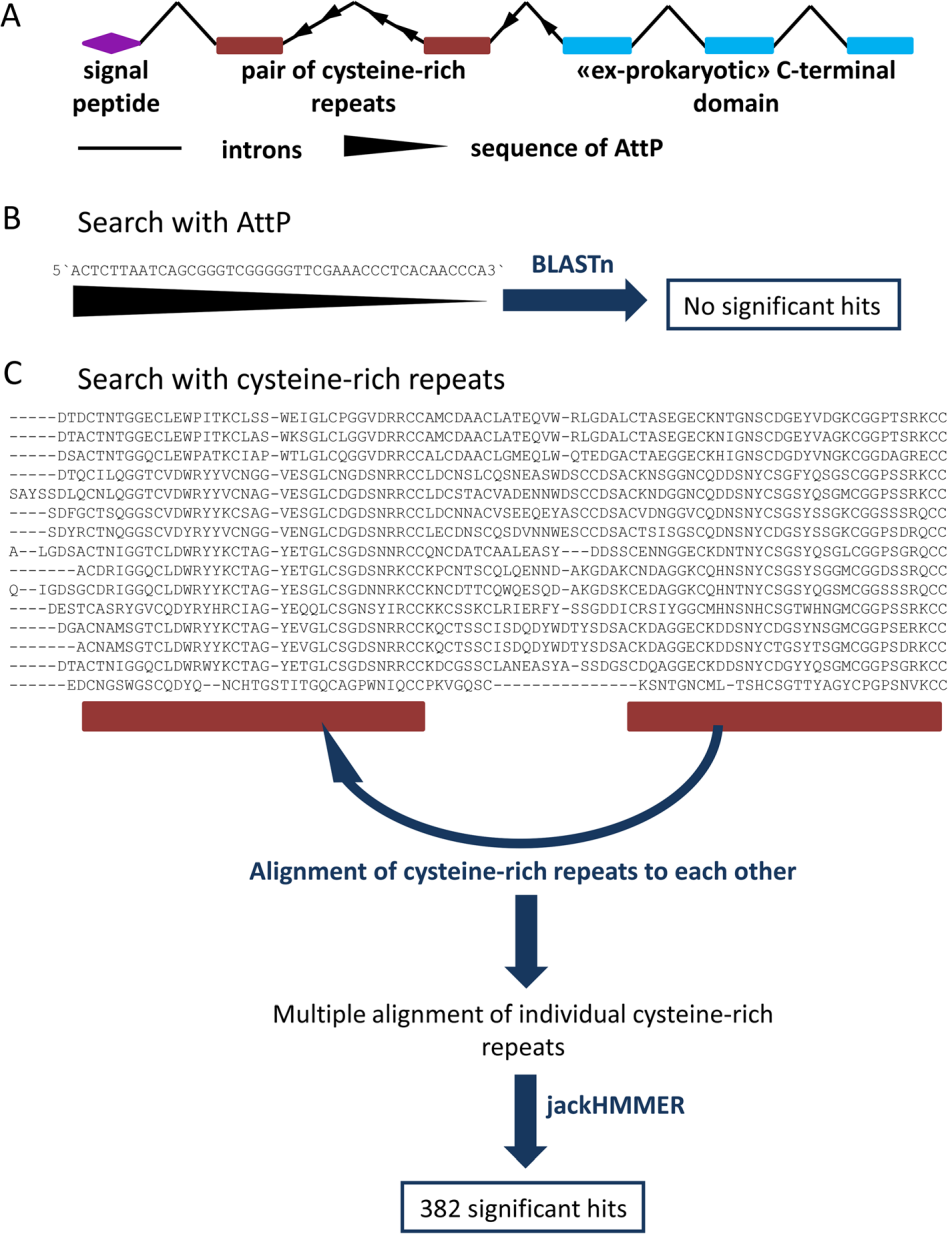
Two strategies of using the sequence of bacteriophage A500 recombination site (AttP) to find potential cases of HGT. **a** The structure of rusticalin-like gene of ascidian *Ciona intestinalis.* Exons are shown as boxes. **b** AttP nucleotide sequence of bacteriophage A500 used as a query for BBLASTn search. **c** Cysteine-rich repeats of the rusticalin and rusticalin-like proteins, used for jackhmmer search

Our approach at finding bacteriophage recombination sites using BLASTn search through all available eukaryotic genomes returned no significant hits (Fig. 1b). Since our previous results indicated that AttP sites lie inside the introns of rusticalin cysteine-rich repeats (Fig. 1a), we switched to using a larger amino acid sequence of cysteine-rich repeat itself for a remote similarity search. For that purpose, we split each cysteine-rich repeat, present in rusticalin and rusticalin-like proteins, into individual cysteine-rich modules. Those modules were aligned to each other in order to obtain a multiple sequence alignment (Fig. 1c). This alignment of individual cysteine-rich modules was used in a remote similarity search by JackHMMER in UniProtKB database. Three iterations of jackhmmer gave a maximum number of hits with a small number of hits losses. It resulted in 382 significant matches with protein sequences (Supplementary material, Table T1) with E-value ranging from 1.4e-207 to 0.0098.

**Fig. 2:**
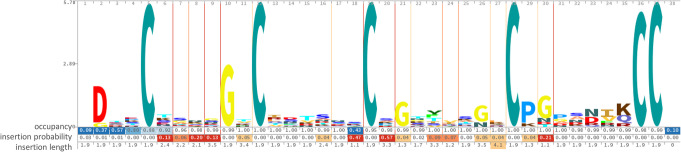
The consensus sequence of a single cysteine-reach repeat depicted with HMM Logo. The relative height of the letter in each position indicates the level of conservation. Y axis – information content (bits). Three lower rows indicate: occupancy, with stronger blue background indicating lower occupancy, insertion probability, and insertion length, with stronger red background indicating higher values. Upper row of numbers indicates the position of the model [42]

**Fig. 3:**
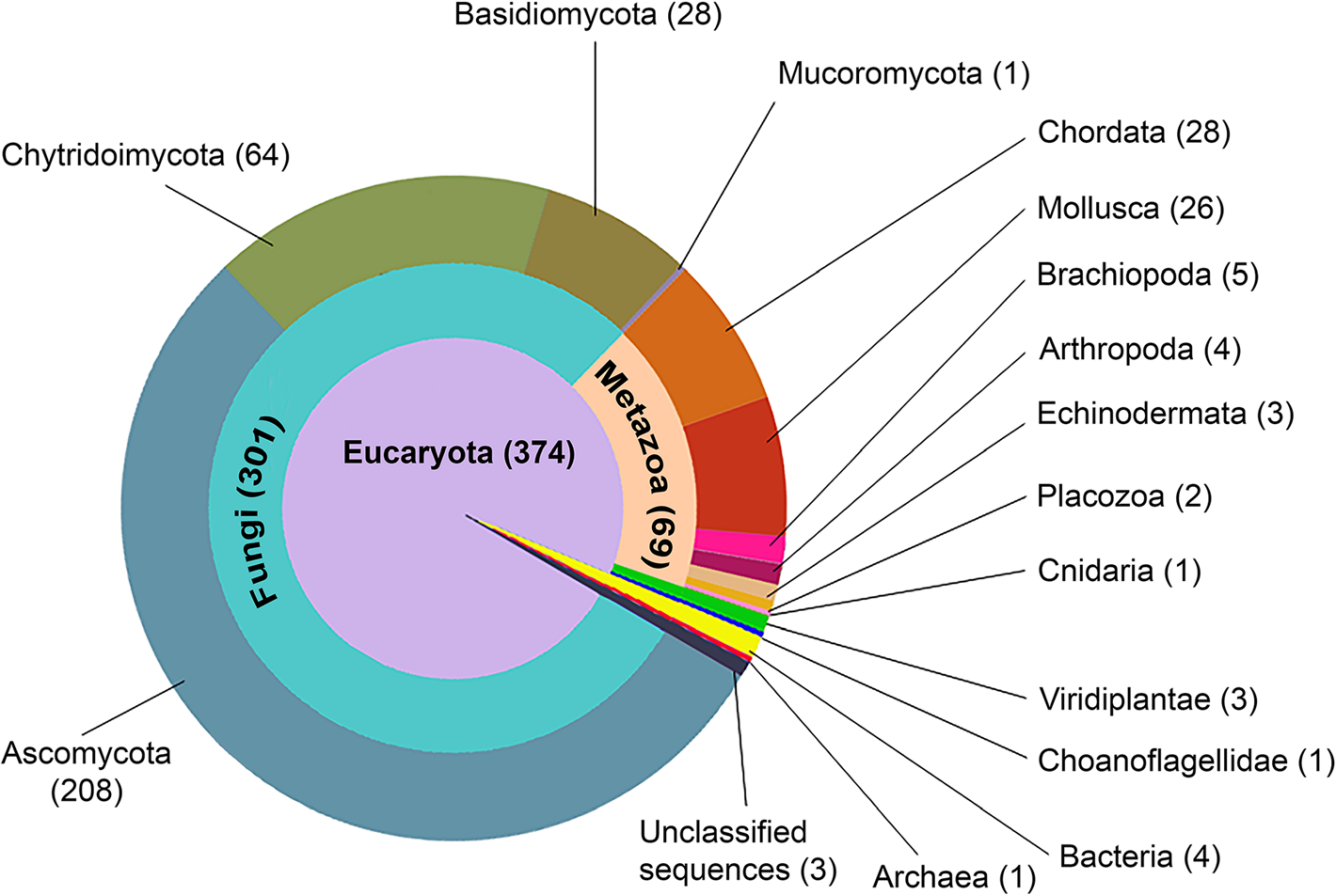
Taxonomic distribution of the proteins containing one or more cystein-rich repeats. Number of related proteins found in each taxonomic group is indicated in the parentheses

Resulting protein dataset was used for future analysis. For all proteins containing cysteine-rich repeats an HMM Logo of an individual repeat was constructed (Fig. 2), showing the level of conservation for each amino acid position. Cysteine pattern appeared to be absolutely conserved. Among the other conservative amino acids there are amino acids with small neutral side chains like glycine (positions 10, 21, 26 and 30) and proline (positions 29 and 31). This sequence was usually present in multiple copies. The taxonomic distribution of the proteins containing one or more cysteine-rich repeats is given in Fig. 3. Such proteins are almost exclusively eukaryotic (374 out of 382): majority of the proteins (301 out of 382) belong to Fungi and only 69 to Metazoa (Fig. 3). The distribution of hits among metazoan taxa is patchy. We found a wide variety of phyla but low abundance of the proteins in each phylum.

### Conserved domains associated with cysteine-rich repeats

**Fig. 4:**
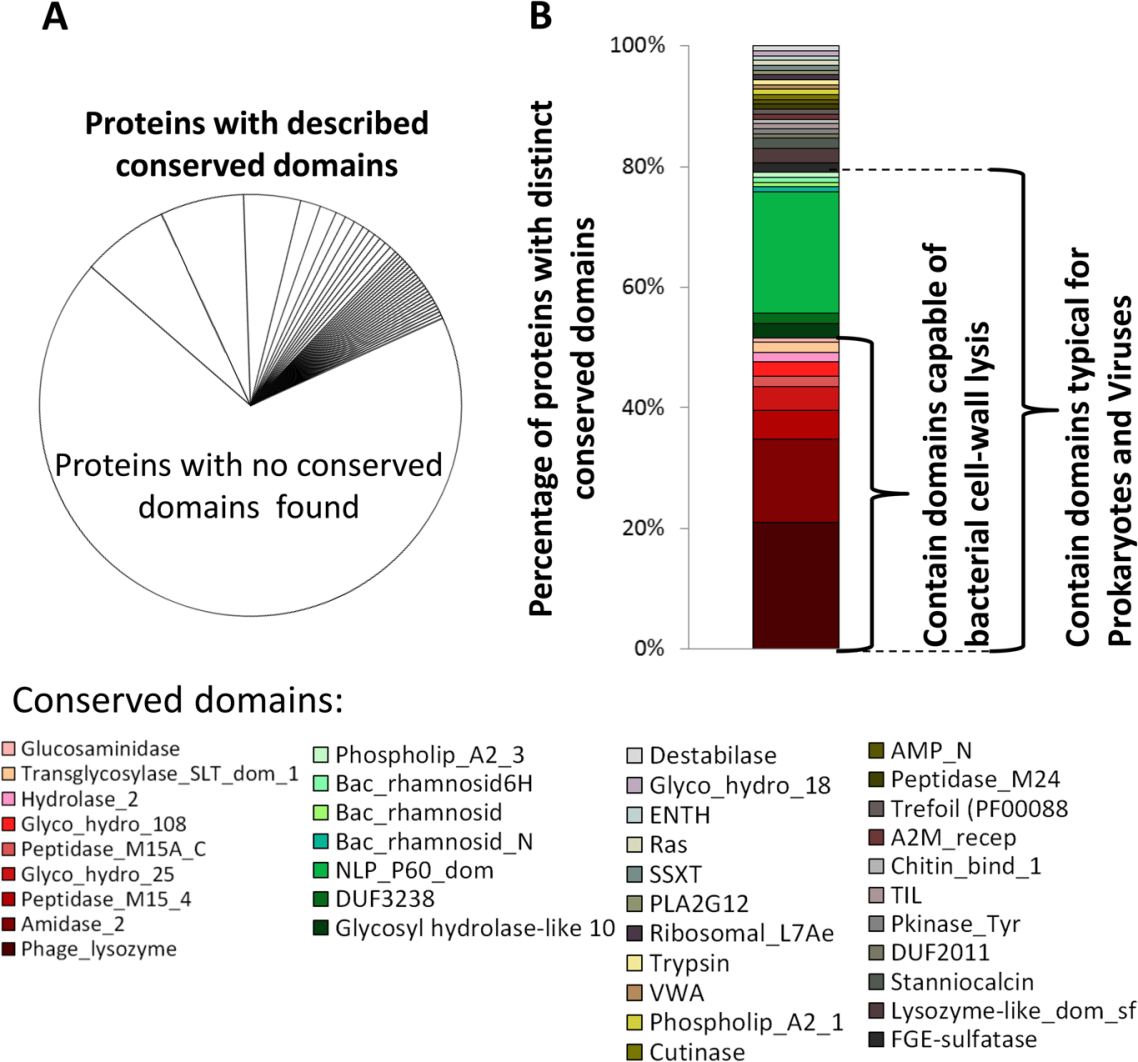
Conserved protein domains associated with cysteine-rich repeats. **a** The presence of conserved domains in proteins containing cysteine-reach repeats, as identified by jackHMMER remote similarity search. **b** Percentage of proteins by function and taxonomic affiliation of conserved domain. The function and confinement to a specific taxon were retrieved from Pfam, InterPro and CAZy databases

Cysteine-rich repeats are usually found as parts of larger proteins. In our study 124 proteins had such repeats present together with other annotated conserved domains (Fig. 4a). These proteins formed a restricted dataset which was used in further analysis. In the other 258 matches cysteine-rich repeats were present, but no associated annotated domains were found nearby. We believe these proteins must be analyzed separately and deserve a dedicated study.

Among 124 proteins containing cysteine-rich repeats and predicted conserved domains, in 20% (26 proteins) such repeats were associated with phage-lysozyme (PF00959), in 14% (17 proteins) with zinc amidase (PF01510) and in 5% (6 proteins) with Peptidase M15 (PF13539). In a few other proteins the cysteine-rich repeats were associated with other domains (Fig. 4b). For each conserved domain we screened its global species distribution with automatic Pfam description [43]. We identified a total of 16 different domains that can be classified as typical for prokaryotes or bacteriophages and they are present in 79% (98 out of 124) of proteins with characterized conserved domains. Remaining 21% of proteins containing annotated domains had no bias towards prokaryotes in their taxonomic distributions. The search for physiological functions of the possible prokaryotic or viral domains found revealed that nine are bacterial cell-wall hydrolyzing enzymes and they are present in 51% of proteins (63 out of 124) (Fig. 4b). Other seven domains are either not involved in cell-wall destruction, or their functions are unknown. Nevertheless all proteins containing domains typical for prokaryotes or bacteriophages may be considered as candidates for being originated through HGT process.

### New case of HGT

**Fig. 5:**
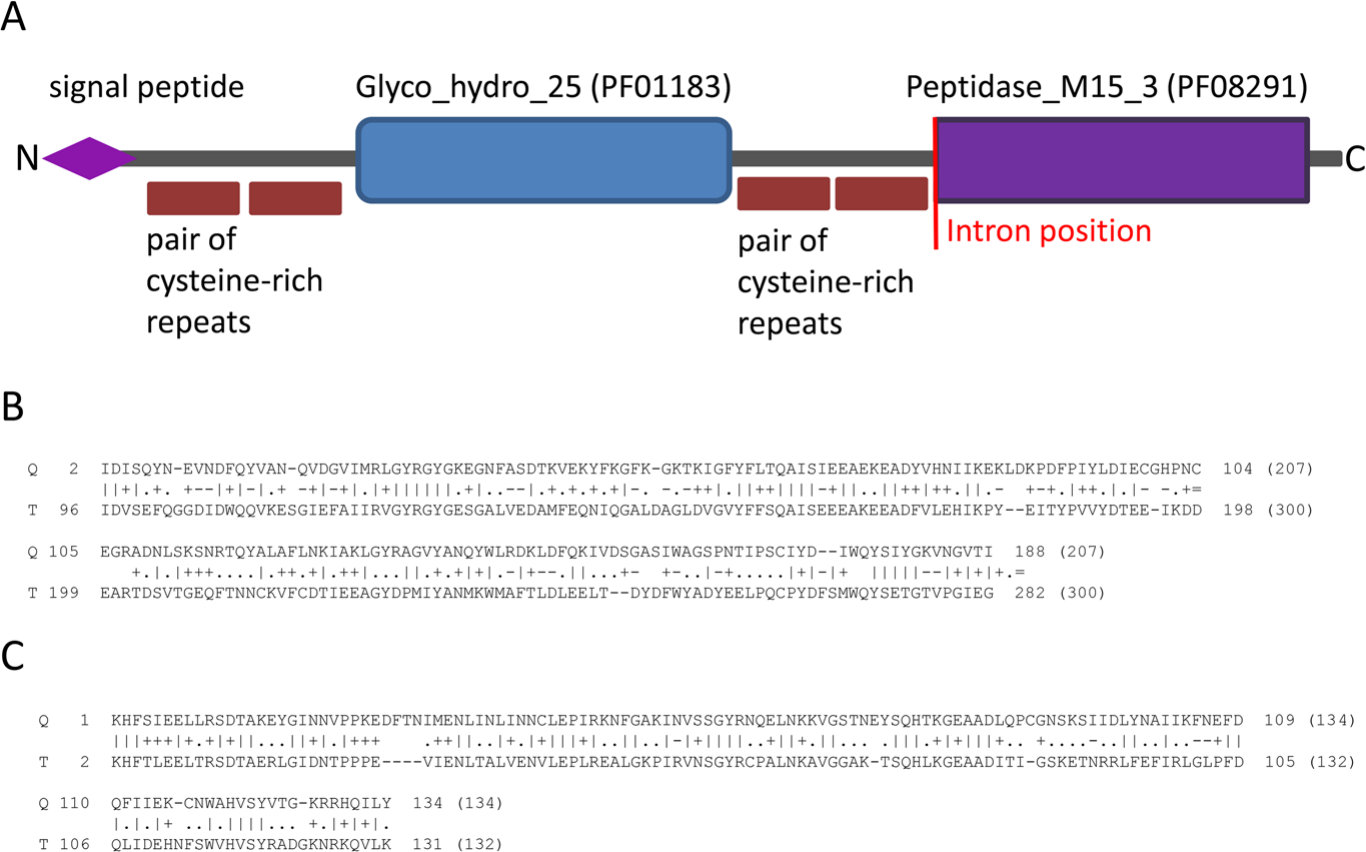
Protein of a fungus *Neocallimastix californiae,* containing two typical prokaryotic domains. **a** Protein structure, showing Glycoside hydrolase and Peptidase M15 domains, each accompanied by a pair of cysteine-rich repeats. **b** Alignment of Glycoside hydrolase domain with bacterial *Lachnoclostridium* sp. lysozyme. The sequence identity is 35%. **c** Alignment of Peptidase M15 domain with bacterial *Bacteroides clarus* peptidase. The sequence identity is 50%

In a restricted protein dataset we identified several HGT candidates based on the description of conserved domains. In order to identify the potential records of multiple transfer events we chose proteins with more than one predicted conserved domains. Two proteins from the fungus *Neocallimastix californiae* (UniProt ID A0A1Y2AHN7 and A0A1Y2FMX2) each contained a pair of different predicted domains typical for prokaryotes: Glucosaminidase (PF01832) coupled with Endopeptidase (PF000877) or Glycoside hydrolase (PF01183) coupled with Peptidase M15 (PF08291) respectively (Supplementary Table 1). We consider these domains “ex-prokaryotic”, i.e. originating from prokaryotic ancestor by means of horizontal transfer (HGT). In order to check if the presence of cysteine-rich repeats can predict proteins resulting from HGT, we chose A0A1Y2FMX2 protein for further analysis. This protein contains Glycoside hydrolase domain (PF01183) and Peptidase M15 domain (PF08291), each of which is accompanied by a pair of cysteine-rich repeats at its N-terminal side. A common eukaryotic signal peptide is predicted at its N-terminus from Met1 to Ala25. The DNA sequence of the corresponding gene contains one intron following the second pair of cysteine-rich repeats and preceding the Peptidase M15 domain (Fig. 5a), a feature typical for eukaryotic sequences. Based on HHblits search, amino acid sequence of Glycoside hydrolase domain has three nearest relative sequences from the genus *Piromyces* – another genus from the same family Neocallimastigaceae. The fourth most significant hit (E-value: 2.0E-28) was with bacterial *Lachnoclostridium* sp. lysozyme (Fig. 5b). In other words, three of the sequences related to Glycoside hydrolase domain are found among other fungi, while the next most similar sequence occurs not in a phylogenetically close taxon, but in a very distant group belonging to prokaryotes. High percentage of the identical amino acids (35%) and a very low E-value (2.0E-28) suggest that these sequences are related. At the same time, for Peptidase M15 domain of the same *N. californiae* protein, the closest significant hit (E-value: 2.6E-33) was with bacterial *Bacteroides clarus* peptidase (Fig. 5c). The proportion of identical amino acids in that case was 50%. Both low E-value and high identity rate also indicate that the fungal and bacterial sequences should be related. This strongly suggests that both Glycoside hydrolase and Peptidase M15 domains in the coding sequences of *N. californiae* protein might have originated from a prokaryotic ancestor.

The same logic is applicable for the protein A0A1Y2AHN7 containing Glucosaminidase (PF01832) coupled with Endopeptidase (PF000877). Those two domains might have come from two independent events of HGT, but they also could have been transferred as a single DNA fragment from a single prokaryotic donor organism.

## Discussion

The only mechanism of prokaryote-to-eukaryote DNA transfer established so far involves a bacterial pathogen donor and a plant host as an acceptor. In this case DNA is delivered into the nucleus and integrated into the chromosome by the host DNA repair machinery, summarized in [7]. Another model of HGT involving bacteriophage as a vector of gene transfer from prokaryote donor to eukaryote acceptor was proposed earlier [15, 21, 22, 44, 45]. In particular it have been described that horizontally acquired genes were associated with prophage regions in the donor *Wolbachia* genome [46]. The results of our previous study agree with these findings. The presence of the bacteriophage recombination sites (AttP) next to horizontally transferred genes in eukaryotic genome as well as in bacterial donor genome supports this hypothesis. In the present study we demonstrated that a search for bacteriophage recombination site in eukaryotic genomes can reveal new cases of HGT. Although, since the nucleotide sequence of AttP is too short to get significant hits, we switched to a larger amino acid sequence of cysteine-rich repeats harboring AttP inside its introns. Cysteine-rich repeats happened to be conservative across multiple fungal and metazoan proteins. The similarity between cysteine-rich repeats and β-defensins may suggest that both are involved in immune reaction [47–49]. The distribution of the proteins with cysteine-rich repeats among Metazoa is patchy and not concentrated in any particular phylogenetic group. This disjointed distribution was previously described as a hallmark of horizontally acquired genes [27, 28, 45] and was also used as an instrument to find transposon horizontal transfers [50].

Here we further analyzed domain architecture of the proteins containing cysteine-rich repeats. In 124 cases conserved domains were predicted to accompany cysteine-rich repeats, while for 258 remaining proteins prediction was unsuccessful. This may be due to the low conservation of amino acid sequences between different taxa [51, 52]. It is also possible that other prediction instruments [53] such as Motif Scan (https://myhits.isb-sib.ch/cgi-bin/motif_scan) or MOTIF search (https://www.genome.jp/tools/motif/) would be more sensitive than HMMER annotation we used here. Some of the proteins with no predicted domain architecture belong, nevertheless, to the species with previously described multiple cases of HGT. Examples include the fungi *Pochonia chlamydosporia* probably harboring 100 kb region of foreign DNA [54], *Fusarium oxysporum* [55] and *Metarhizium majus* [56]. Cysteine-rich repeats found in these species proteins might provide a bacteriophage dependent mechanism for such HGT events.

In a restricted dataset of the 124 proteins containing predicted conserved domains, we screened the taxonomic distribution of each domain using Pfam and InterPro databases. Even though cysteine-rich repeats themselves are found almost exclusively in eukaryotic proteins, their associated domains, which we were able to identify, were often typical for prokaryotes or bacteriophages. Such associations constituted 79% of the hits in our restricted dataset. Based on this high incidence, we hypothesize that such domains are originated through HGT. Moreover, the cutinase domain, which is uniformly present in prokaryotes and eukaryotes, was likely transferred laterally from Bacteria to Fungi [27]. Thus, we may even underestimate the proportion of HGT domains in our dataset.

Some domains associated with cysteine-rich repeats were described earlier as HGT participants. For example, phage lysozyme was found to be horizontally transferred in bivalve mollusks genome [57] and Glycoside hydrolase domain was probably inserted independently into multiple genomes: in Bacteriophages, Archaea and in three clades of Eukarya [44]. We found Peptidase M15 domain in Fungi and Metazoa (*Trichoplax adhaerens*) and Amidase_2 domain in tree lineages of Metazoa (Chordata, Molluska and Arthropoda) (Supplementary Table T1). In those cases we can also hypothesize independent transfer events.

Two of the proteins in our dataset happened to contain two different predicted domains with suggested “ex-prokaryotic” origins. Such unusual domain architecture leads us to assumption of chimeric origin of these proteins, where the coding sequences of individual domains could have been inherited from prokaryote donors. In many cases of HGT among bacteria, it is the protein domains rather than whole genes, considered as units of transfer [58, 59]. According to our results, two domains of the fungal protein A0A1Y2FMX2 show significant similarity (E-value: 2.0E-28 and 2.6E-33) to bacterial sequences. At the same time no other closely related proteins were found among other taxa. Glycoside hydrolase domain has the putative homolog sequence in the genome of bacteria *Lachnoclostridium* sp., while the most similar sequence to Peptidase M15 domain was found in the genome of bacteria *Bacteroides clarus.* Each of these domains in a protein sequence was accompanied by a pair of cysteine-rich repeat. It is worth mentioning that the genus *Lachnoclostridium* belongs to the phylum Firmicutes, while the genus *Bacteroides* belongs to the phylum Bacteroidetes. Thus, two probable bacterial donors occupy very distant phylogenetic positions [60]. This fact suggests that there might have been two independent HGT events which created a protein with two “ex-prokaryotic” domains.

Both Glycoside hydrolase and Peptidase M15 are enzymes capable of bacterial cell wall lysis [61–64]. We also found a bias towards bacterial cell-wall destruction among the functions of the other predicted domains. Cysteine-rich repeats might serve as antimicrobial peptides penetrating bacterial cell wall in conjunction with lytic enzymes. Such conjunction may even give the organism an immediate selective advantage in antibacterial defense.

Many other described cases of HGT involve a variety of enzymes [59, 65, 66] covering a broad range of metabolic functions [6], whereas proteins predicted in our study as HGT cases are largely supposed to be cell-wall lytic enzymes. Taking into account that bacteriophages use cell-wall lytic enzymes during the replication cycle [67, 68], we hypothesize bacteriophage involvement as a vector of transfer. In this case a new foreign protein would be carried not only as a sequence residing in viral genome but also might serve as a functional enzyme for a prolonged period of time before its horizontal transfer into a eukaryotic cell. Numerous cell-wall destruction enzymes, found in this study, might serve as indirect evidence that bacteriophage was a transition step in gene transfer. We previously hypothesized that the sequence of bacteriophage recombination site (AttP), located inside cysteine-rich repeats, can facilitate a type of HGT which involves bacteriophage as a vector of gene flow. Among the proteins described in the present study, some contain domains typical for bacteriophages, but no direct homology was found. This is probably due to the fast evolutions of viral genomes [69, 70] which can mask the similarity of related proteins [71]. Nevertheless, cysteine-rich repeats can serve as an instrument to find new cases of prokaryote to eukaryote HGT. We also demonstrated that a split of amino acid sequence according to the predicted domain borders may help to infer the ancestry for each domain separately and detect HGT cases.

## Conclusions

Cysteine-rich repeats in eukaryotic proteins are usually accompanied by conserved domains typical for prokaryotes or bacteriophages. Those chimeric proteins probably represent multiple independent HGT events from prokaryotes to eukaryotes. The explanation of this phenomenon may lie in the presence of bacteriophage recombination site, which potentially facilitates HGT, inside the coding sequence of the cysteine-rich repeats.

## Methods

### Constructing the dataset of the proteins containing cysteine-rich repeats

In order to find HGT candidates, we searched all eukaryotic genomes present in the nr, est and TSA GenBank databases for the nucleotide sequence of bacteriophage AttP using BLASTn. Since it provided no significant hits, we processed amino acid sequences of cysteine-rich repeats for subsequent remote similarity search instead. Cysteine-rich repeats are present in rusticalin and rusticalin-like proteins as a pair [41]. The borders of repeats in this amino acid sequence were predicted using REPRO [72]. According to those borders, each member of repeat pair was split into two individual cysteine-rich modules. At the next step we aligned all modules of all rusticalin-like proteins with MUSCLE 3.8.31 [73]. Multiple sequence alignment of individual cysteine-reach modules was subjected to remote similarity searches using online version of HMMER 3.1b2 jackhmmer [74] in UniProtKB v.2017_08 protein database. The resulting list of hits became a raw dataset of the proteins containing cysteine-rich repeats.

### Conserved domains analysis

Taxonomic distribution of proteins, their domain architecture and number of cysteine-rich repeats per protein were defined by jackhmmer in the HMMER 3.1b2 package [74]. The same package was used to automatically assign every individual domain to a specific protein family. Domains were considered conserved when they matched the existing Pfam 32.0 database entries [43, 75]. We hypothesized that taxonomic distribution of a domain might differ from the taxonomic distribution of a protein. Thus, confinement of a conserved domain to a specific taxon was derived from the species distribution information in a Pfam 32.0 database (http://pfam.xfam.org/) [43, 75]. A domain was considered typical to prokaryotes or viruses if more than three quarters of carrier species belonged to those groups. The functions of the discovered conserved domains as a possible cell-wall hydrolytic enzyme was inferred based on the information from Pfam 32.0, InterPro 74.0 (http://www.ebi.ac.uk/interpro/) [76] and CAZy 2019/03/20 (http://www.cazy.org/) [77] databases, as well as the original literature [64].

### Search for homologous sequences

One of the proteins from the fungus *Neocallimastix californiane* (A0A1Y2FMX2) was thoroughly analyzed. Signal peptide position in amino acid sequence was predicted with SignalP5.0 [78]. Intron position in the corresponding genomic sequence was retrieved from the whole genome shotgun sequence of *N. californiane* (GenBank MCOG01000004.1, positions 1,277,192–1,279,411). Positions of the conserved domains were predicted using InterPro 74.0. Finally, amino acid sequences of the individual domains, cut out of the whole protein sequence, were subjected to a remote homology search in the Uniclust30_2018_08 database [79] using HHblits 3.2.0 [80, 81] of the MPI Bioinformatics Toolkit web site (https://toolkit.tuebingen.mpg.de/).

## Supplementary information

**Additional file 1: Supplementary Table T1.** Results of three iterations of jackhammer search. Proteins containing cysteine-rich repeats.

## Data Availability

All data analyzed during this study are publicly available at GenBank (https://www.ncbi.nlm.nih.gov/genbank/), UniProtKB (https://www.uniprot.org/), Pfam (https://pfam.xfam.org/), InterPro (https://www.ebi.ac.uk/interpro/) and CAZy (http://www.cazy.org/) databases. All data generated during this study are included in this published article [and its supplementary information files].

## Abbreviations

HGT: Horizontal gene transfer
AttP: Bacteriophage recombination site
